# Optimization of Skewed Data Using Sampling-Based Preprocessing Approach

**DOI:** 10.3389/fpubh.2020.00274

**Published:** 2020-07-16

**Authors:** Sushruta Mishra, Pradeep Kumar Mallick, Lambodar Jena, Gyoo-Soo Chae

**Affiliations:** ^1^School of Computer Engineering, Kalinga Institute of Industrial Technology, Deemed to be University, Bhubaneswar, India; ^2^Department of Computer Science and Engineering, Siksha ‘O' Anusandhan Deemed to be University, Bhubaneswar, India; ^3^Division of Information & Communication, Baekseok University, ChePonan-si, South Korea

**Keywords:** data skewing problem, machine learning, best first search, KNN algorithm, SMOTE, SpreadSubSampling, F-score

## Abstract

In the past few years, classification has undergone some major evolution. With a constant surge of the amount of data gathered from different sources, efficient processing and analysis of data is becoming difficult. Due to the uneven distribution of data among classes, data classification with machine-learning techniques has become more tedious. While most algorithms focus on major data samples, they ignore the minor class data. Thus, the data-skewing issue is one of the critical problems that need attention of researchers. The paper stresses upon data preprocessing using sampling techniques to overcome the data-skewing problem. Here, three different sampling techniques such as Resampling, SpreadSubSampling, and SMOTE are implemented to reduce this uneven data distribution issue and classified with the K-nearest neighbor algorithm. The performance of classification is evaluated with various performance metrics to determine the efficiency of classification.

## Introduction

Recently, enormous data is aggregated on a daily basis. Many times, it is observed that such massive data samples are unevenly matched and classified among its classes. As a result, generation of data occurs in a skewed way. This scenario in a data set where samples of data in one class are much higher in comparison to that of the other class is represented as a skewed data set. In this case, the higher data sample class becomes the major class and the class consisting of relatively less data samples is labeled as minor class. As a result of this uneven distribution of data samples, major class is given higher importance than minor class. Hence, the overall performance of machine-learning algorithms is affected, thereby generating inaccurate results. However, in the classification process, both major and minor class samples play a significant role. Subsequently from previous studies, it is observed that this skewed data has a major impact on the performance of machine-learning algorithms (1–3). Besides this, acute knowledge mining from minor class is also a factor considering there are very few data instances (4). This data-skewing concept holds significance, which is applied in several real-time applications such as remote area sensing (5), acute pollution detection (6), risk identification and control (7), and fraud detection and prevention (8). This data skewing can be a critical bottleneck in sensitive applications like in the medical domain in diagnosis of patients where a minor negligence can be dangerous for patients' health. Usually, various classification algorithms that are deployed in predictive analytics consider homogeneity in data partitioning among its classes. It may be a hurdle, thereby degrading the effectiveness of performance of machine-learning models. Apart from this, overhead causes due to rate of occurrence of error in data skewing are unbalanced, creating inconsistency during classification. It may result in classes overlapping for which noisy instances rise, creating more complexity in prediction.

## Sampling

The problem of imbalance aggregation of samples of data may be effectively dealt with by the use of a data preprocessing approach called Sampling. It is used to handle problems of uneven data distribution in a given data set. The prime objective of this sampling approach is to identify and choose a sample of data from the raw unstructured data sets gathered, which represent the overall data records. By deploying this mechanism, a smaller data section can be mapped to the entire data set. Two features that govern the selection of a sample include the size of the data sample and the quality of the sample. Several distinct criteria are there for selecting a sample rather than a complete database, which are as follows:

- It is suited in large data sets, which involves handling several constraints.
- The approach of data filtering and preprocessing is cheap.
- Relative loss in data is least.
- It is robust and dynamically applicable to different data sets.

Basically, there are two ways to deal with Sampling which includes Under-Sampling and Over-Sampling. Under-Sampling is performed on the larger training data set while Over-Sampling is done on minority data records.

**Figure 1:**
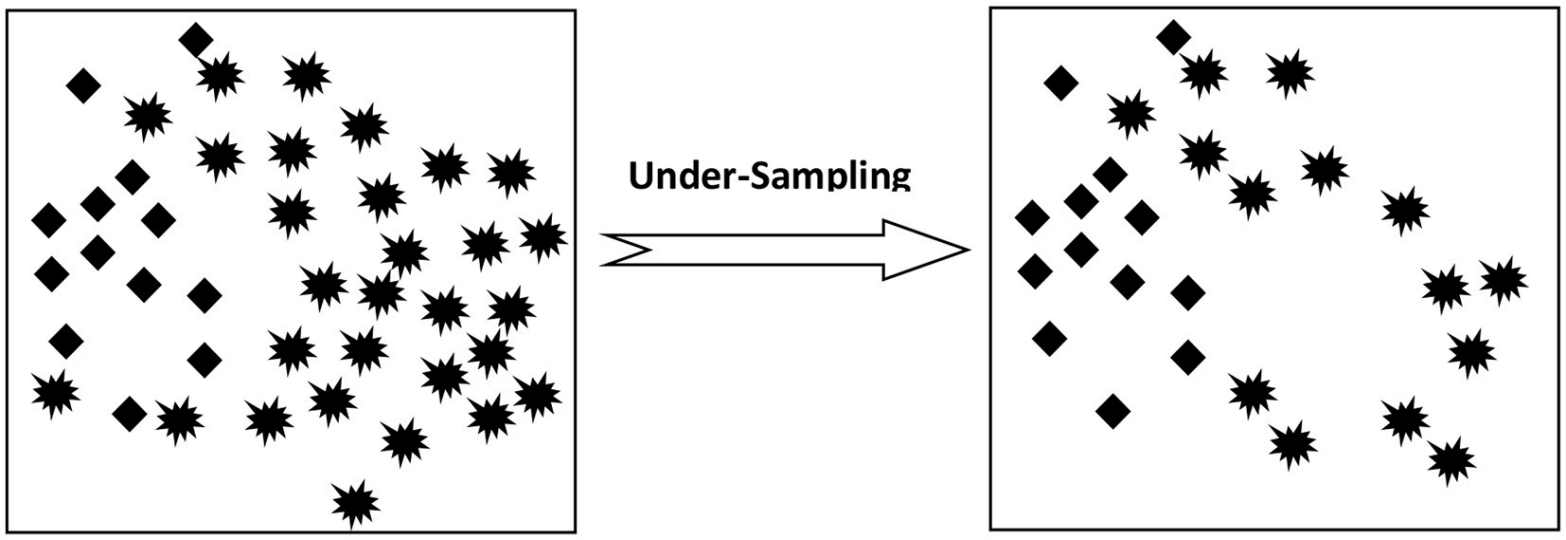
Under-sampling process.

*Under-Sampling*: This approach is applicable to a class belonging to majority data samples. Here, data samples of data are selected and eliminated at random so as to create a balance between both class data as shown in Figure 1.

**Figure 2:**
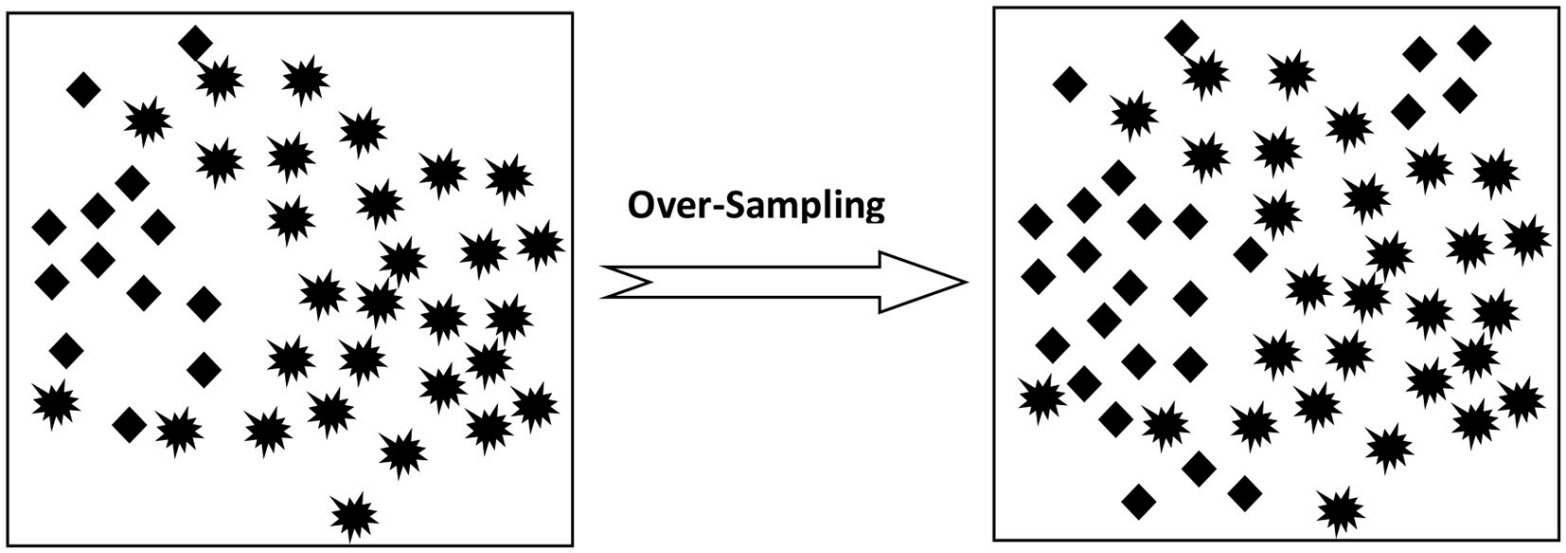
Over-sampling process.

*Over-Sampling:* This procedure is applicable to enhance the size of data sets in the class with fewer instances so as to match it with the class with a larger number of data distributions as shown in Figure 2.

## Related Work

In (9), the authors discussed a method to determine the features on probability density technique by taking a small data set consisting of uneven classes on the ranking of attributes. Nguwi and Cho (10) present a method of attribute selection that represents a weight vector based on a support vector machine. Its basic aim was to remove the unwanted features, thus enhancing the accuracy rate in classification. In (11), a white blood cell classification model based on the nature-inspired approach is developed, and when a comparison analysis with other existing nature-inspired algorithms is done, it was found that the proposed model was extremely fast and precise in the analysis of hematological issues. An efficient machine-learning framework to identify and classify leaves has been proposed in (12). Later, the proposed model was compared with some classifiers like random forest and KNN algorithm. The accuracy rate of the proposed framework outperformed other classifiers in both training and testing data samples. In (13), the significance of the SMOTE method on sparse and heterogeneous data sets is discussed. Here, the decision tree algorithm and naïve Bayes algorithm are used as classifier. In (14), a hybrid model classifier was developed, which is a combination of SMOTE, particle swarm optimization, and RBF (radial-basis function). This hybrid combination resulted in high prediction performance. In (8), authors analyzed the Undersampling and Oversampling effects on backpropagation neural networks and particle swarm optimization. The result highlighted the sensitivity of the PSO algorithm toward the uneven data distribution and training data with minimum number of instances and many attributes. An enhanced and dynamic adaptation of the crow search algorithm is proposed in (15) to make Parkinson disease diagnosis more effectively and accurately. The proposed optimized algorithm yielded an overall prediction accuracy of 100%. In (16), both undersampling and oversampling use a resampling method that defines several parameters in tuning SVM. The unbalanced data aggregation results in a major data shift to the minor class. A study in (17) developed a resampling-based preprocessing technique to address the skewing of unbalanced data sets and classified various types of tumor in patients. Sharma et al. (18) deals with development of an optimized meta-heuristic model for attribute selection to accurately categorize protein structures. The proposed model was experimentally compared with other meta-heuristic algorithms, and it was found that it yielded optimum results than others. Sahoo et al. (19) used the LVQ technique to illustrate and analyze the clustering deviation issue on the breast cancer data set. In (20), the authors developed a class-based method using ant-colony optimization, which is very effective for major classes. The rate of latency was very high in this method, which is a disadvantage. A novel approach is proposed in (21) on the basis of experimental evaluation on polyster compounds reinforced with fiberglass. The PSO algorithm and genetic algorithm were used to predict global optimum, and it was inferred that the convergence of the PSO algorithm was very fast and needed less execution time. A succinct analysis in (22) presented a cluster analysis under-sampling technique. Here, various clusters were formed for partitioning the entire training set. From every cluster, selected data from the major class were chosen according to the proportion of major data to minor data. The research outcome using clustering on under-sampling enhances the rate of classification accuracy, and the model was more robust than others. Highly accurate with least computational cost evolutionary model-based feature optimization techniques are presented in (23) to detect and diagnose lung disease disorders automatically. Sharma et al. (24) presented an optimized and improved feature selection model that is useful in extracting optimum attributes in Parkinson's disease record samples with enhanced efficiency. It achieved an accuracy rate of 95.91% which is much higher than other related algorithms.

## Proposed Work

**Figure 3:**
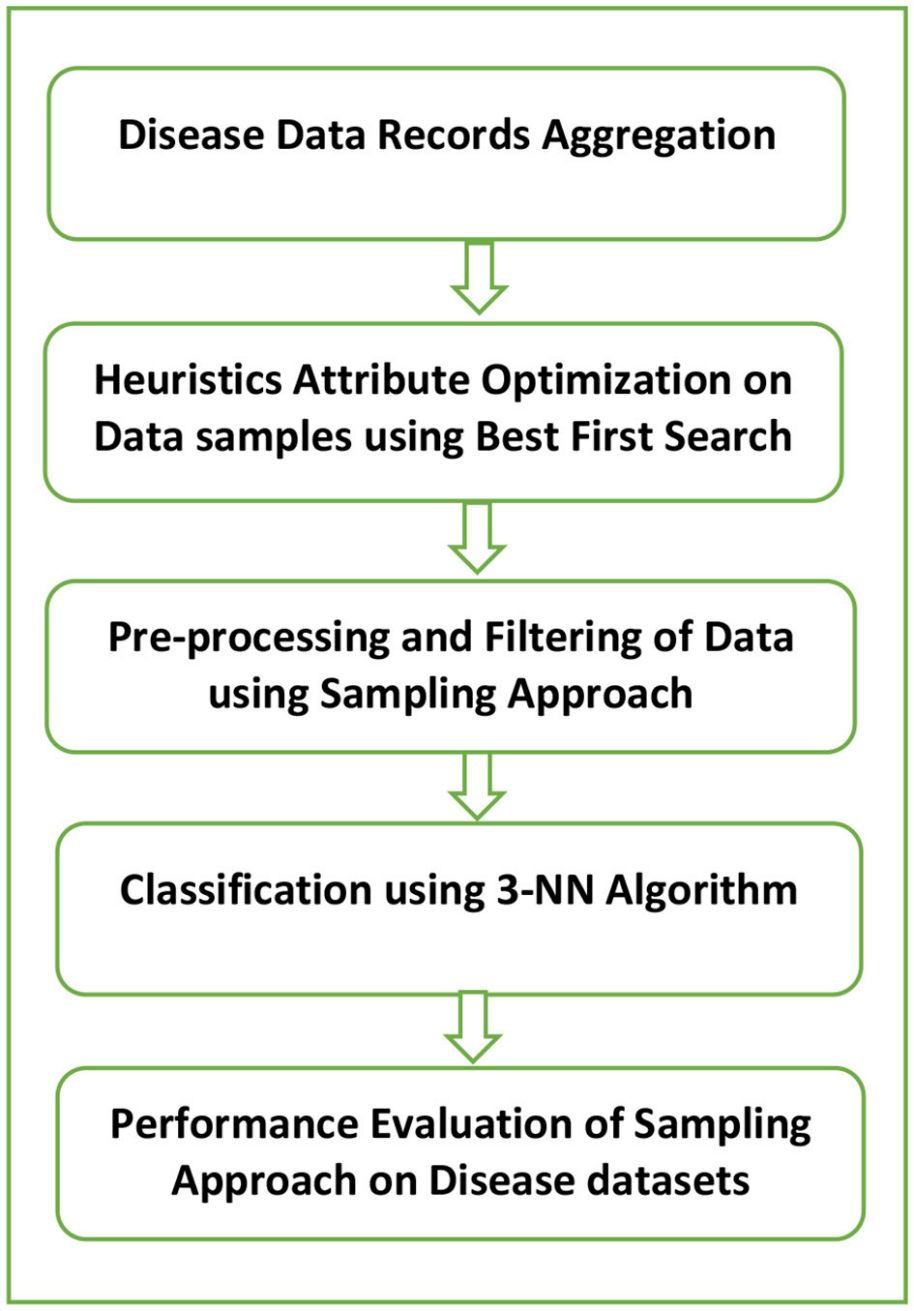
Our proposed hybrid model based on sampling.

The prime purpose of the analysis is to present disease data samples with minimum uneven data issue so that the required adjustment may be done in data segregation of major and minor classes. The developed framework as shown in Figure 3 constitutes a five-phase approach of evaluation of performance with the use of data filtering with sampling. The system model proposed in our work is applicable to the healthcare industry. The data sets under consideration include diabetes, breast cancer, and hepatitis. Initially, the sample disease data sets under consideration were gathered from the UCI repository. In the next step, the best-first search algorithm which is based on heuristic search optimization is applied to the original data samples to remove the less relevant features. It is used in the traversal of the graph to determine one or more goal states, which implements priority queue for its operations. This results in a reduced and optimal data set. Eventually, this optimized data set is implemented with three distinct sampling methods which include SMOTE, Resampling, and SpreadSubSampling. These techniques are useful in varying the sampling data distributions in the already existing data samples where the minority class samples are over-sampled while the majority class samples are under-sampled. This results in a relatively evenly balanced data set. After sampling is performed, the data set undergoes the classification process. The classifier used for this purpose is the K-nearest neighbor (KNN) algorithm where the value of K is 3. It performs classification of a newly arrived unknown data sample based on the Euclidean distance which is used as a similarity measure. It performs classification of new test data on the basis of distance functions used as a similarity metric. During classification, a new unseen data sample is allotted the class label of the most common class among k-closest examples. While classifying a test data, it is allotted a class label of the most common class among 3 closest neighbors. Finally, the effectiveness of the presented model is evaluated by the help of fw vital performance indicators like positive predictive value (PPV), sensitivity, prediction accuracy, F-score, and ROC metric, and the efficiency of the system model is evaluated. The heuristics-based optimization search space technique implemented in our research is best-first search, and the classifier used is the K-NN algorithm where K is taken as 3.

Various sampling techniques used are as follows:

- *SpreadSubSample*: A subsampling filter in which a random subset is processed in order to be fit in memory. In this filter, a maximum spread between the minor and major classes is denoted. For example, the user may specify the class frequency difference to be 2:1. For the subsequent batches, there is no resampling while the batch mode is implemented.
- *SMOTE:* It is a technique specified for Oversampling of the minority class with Random Undersampling of the majority class. The k-nearest minority neighbors are computed in the minority class. Then, some of these neighbors are selected, from which synthetic data samples are extracted which join the minority sample with its chosen neighbors.
- *Resampling:* The objective of Resampling is to add instances to a class. It is done by simply adding instances multiple times to the result data set from the class, which has only a few instances. Suppose one class 2 instance is present then with a resampling with a bias of 1.0, N copies of that instance and N other instances of each other's type for which data is present will be the result. Thus, a random subsample of a data set is produced sampling either with replacement or without replacement.

## Result and Discussion

**Figure 4:**
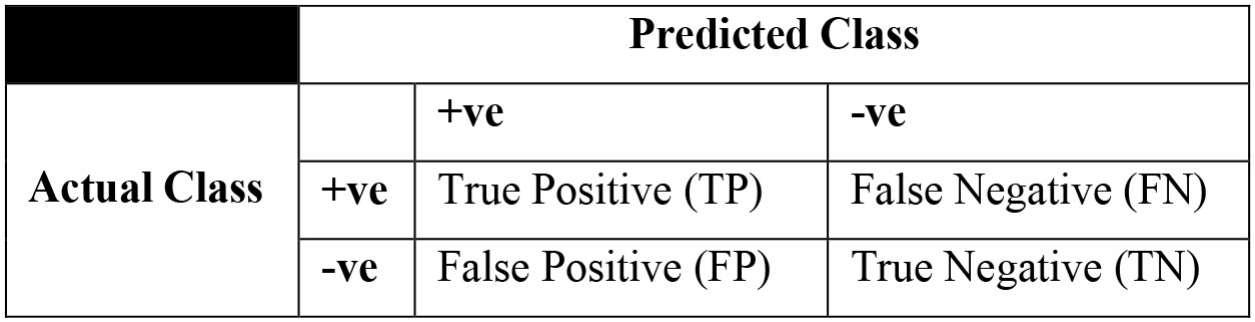
Skeleton of a confusion matrix.

Three sampling approaches which include SMOTE, SpreadSubSample, and Resampling as part of data preprocessing are used in the study. Among the clinical data sets, breast cancer, diabetes, and hepatitis are the data sets under consideration. Various evaluation criteria can be used to determine the effectiveness of machine-learning techniques. However, all evaluation factors may not be useful in dealing with skewed data issue due to the presence of unbalanced data samples in complex data sets. Hence, a confusion matrix may be useful to handle such problem in deriving few important metrics to demonstrate the efficiency of classification. Basic parameters of a confusion matrix are illustrated in Table 1, and a sample confusion matrix is presented in Figure 4.

**Table 1:** Parameters of a confusion matrix.

True positives (TP)	Positive instances correctly identified by classifier
True negatives (TN)	Negative instances correctly identified by classifier
False positives (FP)	Negative instances incorrectly identified by classifier
False negatives (FN)	Positive instances incorrectly identified by classifier

**Figure 5:**
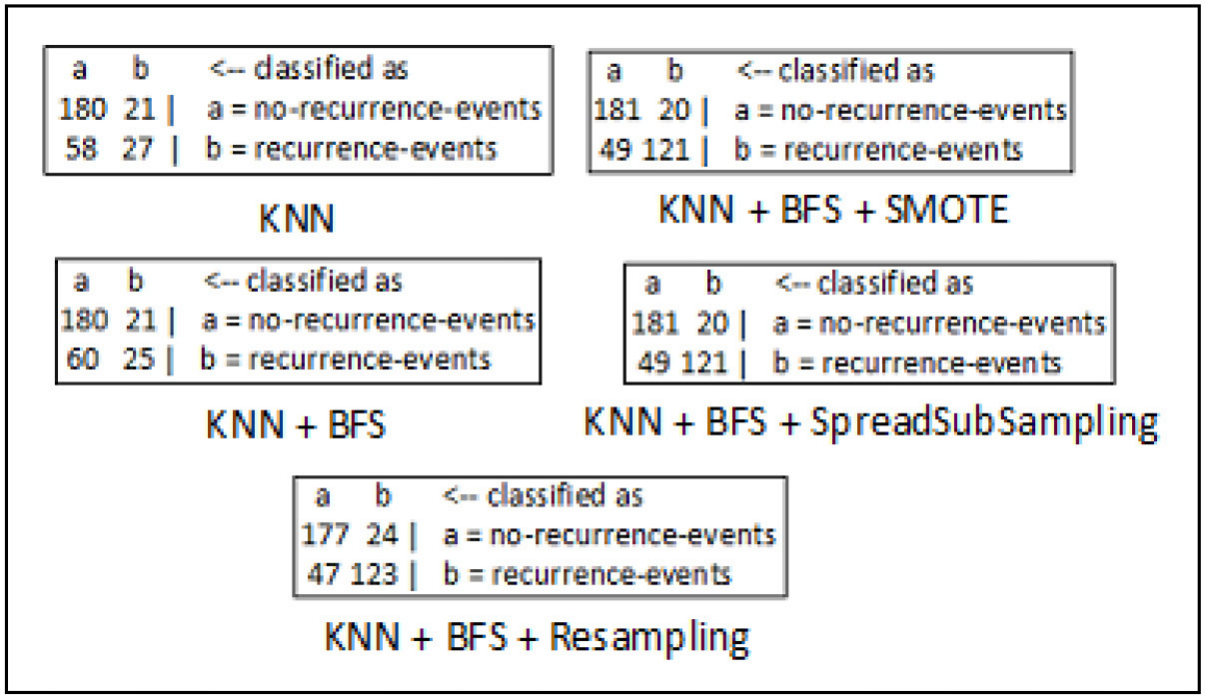
Confusion matrix analysis in breast cancer dataset.

**Figure 6:**
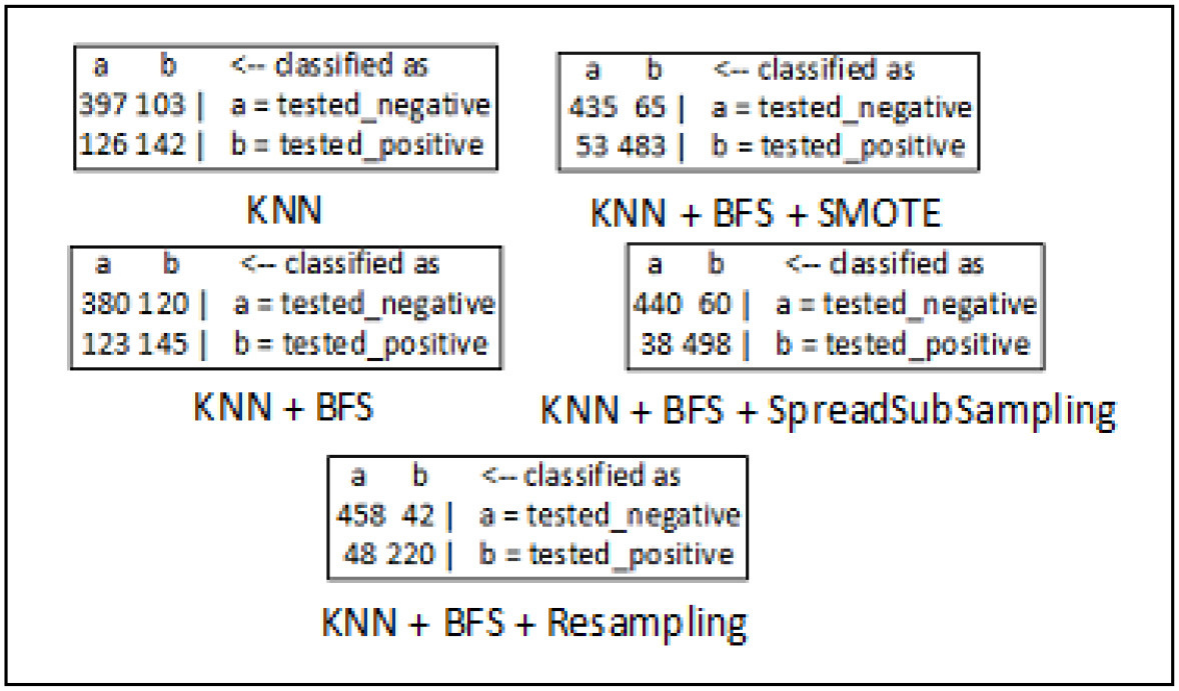
Confusion matrix analysis in diabetes dataset.

**Figure 7:**
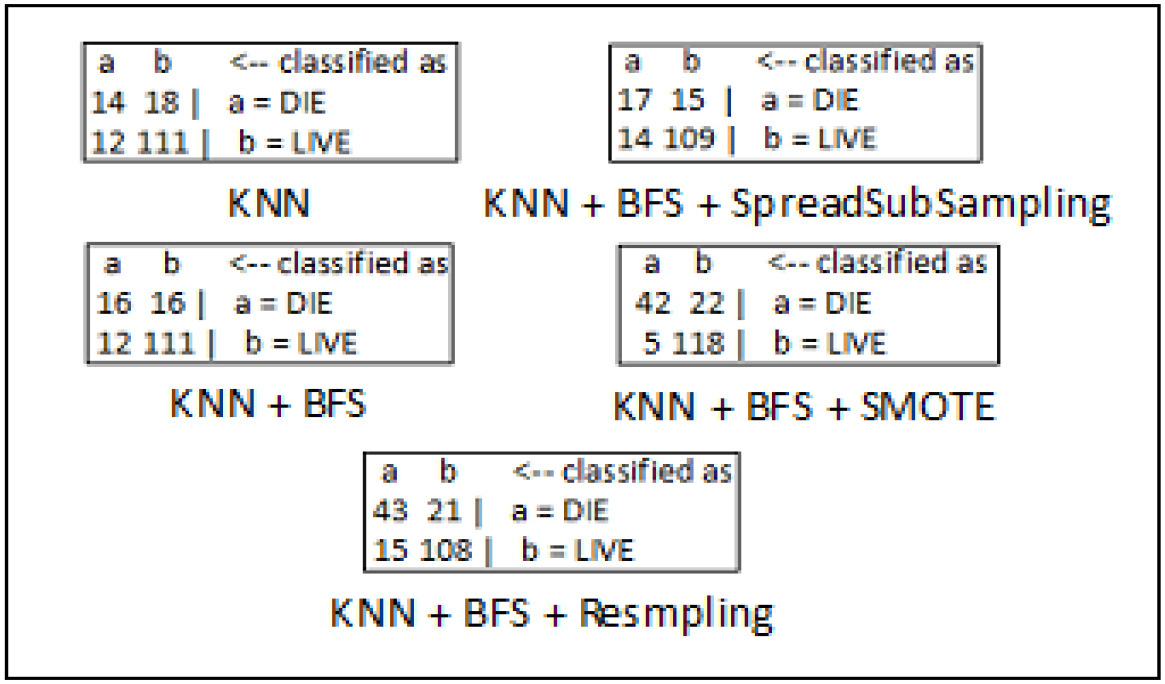
Confusion matrix analysis in hepatitis dataset.

The confusion matrices for breast cancer, diabetes, and hepatitis disease samples are developed using every feasible combination of the KNN classifier, sampling approaches, and best-first search method. The breast cancer data set consists of 286 data records of different patients, and the entire data samples are spread over in two classes which include “*no-recurrence-events*” and “*recurrence-events*”. The confusion matrix of the breast cancer data is seen in Figure 5. The confusion matrix for the diabetes data set is depicted in Figure 6. It has 786 data samples collected from different patients. “*tested_negative*” and “*tested_positive*” are the two classes under consideration in the diabetes data. As observed in Figure 7, “*DIE*” and “*LIVE*” are the two distinct class labels for the hepatitis data set constituting 145 unique samples of patients.

Prediction accuracy forms the basis of classification, which represents the frequency of accurate predictions among all predictions made. Equation 1 denotes the prediction rate of accuracy in terms of confusion matrix.

(1) $$\begin{array}{l} \text{Prediction Accuracy}=\frac{\text{TP}+\text{TN}}{\text{TP}+\text{TN}+\text{FP}+\text{FN}} \end{array}$$

However, at times, prediction accuracy alone is not sufficient to determine the effectiveness of prediction. There are some other equally vital metrics to gauge the prediction performance in skewed data sets such as positive predictive value, sensitivity, and F-score. The positive predictive value denotes the probability of relevance of a randomly chosen data sample from the entire data set.

(2) $$\begin{array}{l} \text{Positive Predictive Value}=\frac{\text{TP}}{\text{TP}+\text{FP}} \end{array}$$

Sensitivity represents the chance of a relevant data sample chosen at random to be extracted in a search.

(3) $$\begin{array}{l} \text{Sensitivity}=\frac{\text{TP}}{\text{TP}+\text{FN}} \end{array}$$

These two metrics may not be so useful in determining the superiority of algorithmic performance in the machine-learning task. For example, if one classification model offers high precision value and low recall value than other models, then it is tough to determine the best model among all. In these scenarios, another evaluation metric named F-score is used. This F-score metric computes the balanced mean value in between recall and precision. The efficiency of a classifier is directly proportional to the value of this F-score metric.

(4) $$\begin{array}{l} \text{F}-\text{score}=\frac{2\text{TP}}{2\text{TP}+\text{FP}+\text{FN}} \end{array}$$

The effectiveness of our proposed model can be evaluated by several performance parameters like sensitivity, positive predictive value (PPV), F-score, and prediction accuracy rate. In case of breast cancer, it is sharply observed that data preprocessing with SMOTE and SpreadSubSample methods yields much better results in terms of the performance metrics taken into account. While with the diabetes data set the SpreadSubSample sampling technique performs relatively better than SMOTE and Resampling methods. With hepatitis data, Resampling and SMOTE methods outperform the SpreadSubSample method. Therefore, it can be clearly observed that the data skewing issue is reduced to a large extent by using the sampling approach and thereby fruitful in balancing the uneven data sets. It leads to more optimal performance with data preprocessing using sampling techniques rather than performing classification without sampling methods. The evaluation result analysis is summarized in Table 2.

**Table 2:** Analysis of different sampling techniques on sample disease data sets yielding optimum performance.

**Healthcare data set**	**Evaluation metric**	**Optimum value**	**Sampling method**
Breast cancer	PPV	0.814	SMOTE and SpreadSubSampling
	Sensitivity	0.820	SMOTE and SpreadSubSampling
	F-score	0.812	SMOTE and SpreadSubSampling
	Prediction accuracy	0.814	SMOTE and SpreadSubSampling
Diabetes	PPV	0.921	SpreadSubSampling
	Sensitivity	0.906	SpreadSubSampling
	F-score	0.905	SpreadSubSampling
	Prediction accuracy	0.884	SMOTE and SpreadSubSampling
Hepatitis	PPV	0.839	Resampling
	Sensitivity	0.837	Resampling
	F-score	0.849	SMOTE
	Prediction accuracy	0.867	SMOTE

## Conclusion

The issue of skewed data is a challenging area, which needs to be handled effectively when dealing with time-specific applications like disease diagnosis in the medical domain. Precise disease diagnosis of patients is a very critical task which requires a high level of accuracy. Our paper has presented the data skewing issue and has demonstrated the use of the data-preprocessing approach by implementing some vital sampling techniques on healthcare disease data sets. Upon implementation of sampling techniques with the KNN classifier on disease data sets, it was observed that the data skewing issue was significantly minimized thereby a more balanced data set is the result.

In this work, sampling techniques like SMOTE, SpreadSubSampling, and Resampling are used. SMOTE was projected as an oversampling method and SpreadSubSampling was used as an under-sampling method for balancing data samples. Though there are no unified norms for class balancing, the study can infer that the classification using the sampling approach generates an optimum result than going in alone. Therefore, it may be concluded that data preprocessing with the sampling approach offers an ideal option to avoid skewing of data samples and is thus beneficial for an effective and accurate disease diagnosis.

## Data Availability Statement

Publicly available datasets were analyzed in this study. This data can be found here: https://archive.ics.uci.edu/ml/index.php.

## Ethics Statement

Ethical review and approval was not required for the study on human participants in accordance with the local legislation and institutional requirements. Written informed consent for participation was not required for this study in accordance with the national legislation and the institutional requirements.

## Author Contributions

SM formulated the concept idea, proposed methodology, and helped in implementation. PM helped in prototype design methodology and implementation work. LJ helped in result analysis work and in documentation work. G-SC helped data interpretation, editing and drafting the manuscript. All authors contributed to the article and approved the submitted version.

## Conflict of Interest

The authors declare that the research was conducted in the absence of any commercial or financial relationships that could be construed as a potential conflict of interest.
